# Exercise and the Heart: Benefits, Risks and Adverse Effects of Exercise Training

**DOI:** 10.31083/j.rcm2403094

**Published:** 2023-03-23

**Authors:** Nilanka N Mannakkara, Gherardo Finocchiaro

**Affiliations:** ^1^Cardiothoracic Centre, Guy's and St. Thomas' Hospital, SE1 7EH London, UK; ^2^Department of Biomedical Engineering and Imaging Sciences, King's College London, SE1 7EH London, UK; ^3^Cardiovascular Sciences Research Centre, St George's University of London, SW17 0RE London, UK; ^4^Cardiovascular Research Centre, Royal Brompton and Harefield NHS Foundation Trust, SW3 6NP London, UK

**Keywords:** exercise, cardiovascular, sudden cardiac death, cardiomyopathy

## Abstract

Exercise has multiple health benefits and reduces cardiovascular morbidity and mortality. Regular exercise decreases the burden of cardiovascular risk factors and improves prognosis in several cardiac conditions. Despite these premises, sudden cardiac death (SCD) during sports may occur in apparently healthy athletes who perform at the highest levels. Accurate identification and prompt treatment of individuals at risk may reduce the burden of SCD. A possible cardiotoxic effect of intense exercise has been recently postulated, however this is still matter of controversy as causal relationships are often difficult to establish taking into account multiple confounders. Exercise is safe for the majority, even with cardiovascular disease. In this review, we focus on exercise and sports, discussing their benefits and risks and exercise recommendations for healthy individuals and those with cardiovascular disease.

## 1. Introduction

Physical exercise is a cornerstone of cardiovascular disease prevention. Sports are widely practiced and prominent in popular culture, with elite athletes often much revered. Several studies have demonstrated lower all-cause mortality [1, 2] and reduced incidence of cardiovascular diseases [3, 4], cancer [5, 6] and metabolic conditions [7, 8] in individuals who engage in regular exercise. Exercise has been shown to decrease the burden of risk factors, including hypertension [9, 10], diabetes mellitus and glucose intolerance [8, 11]. It is therefore strongly advocated by the World Health Organization and in international guidelines for primary and secondary prevention of cardiovascular disease [12, 13, 14].

However, despite the aforementioned positive effects, exercise can be dangerous in individuals with certain underlying cardiac conditions and potentially precipitate sudden cardiac death (SCD). Moreover, the effects of long-term high intensity exercise are not well understood, with some studies suggesting a possible deleterious effect. In this review article, we will discuss the cardiovascular effects of exercise, its benefits and potential hazards.

## 2. Exercise and its Benefits

### 2.1 Definitions and Classification

Physical exercise is commonly defined as “a subset of physical activity that is planned, structured and repetitive, and has as a final or an intermediate objective the improvement or maintenance of physical fitness” [15].

Exercise can be classified according to its type as aerobic or endurance exercise, such as running or cycling, which involves moving large muscle groups for a sustained period of time and requires an increase in heart rate or respiration to meet oxygen demands, or as resistance training (muscle-strengthening activity) that involves using force to produce body movements that strengthen muscles. Exercises that tend to involve short, intense bursts of activity, such as in sprinting or high-intensity interval training may be classified as anaerobic exercise and utilise energy independent of metabolic pathways reliant on oxygen delivery, for example via glycolysis, causing an increase in lactate [16].

Exercise may be further classified according to its static (isometric) and dynamic (isotonic) components [17], which determine the type of workload exerted on the cardiovascular system and its potential effects. Static exercise involves the use of high intramuscular force with minimal joint movement or change in muscle length. It is graded (I to III) by estimated percentage of maximal voluntary muscle contraction and typically results in an increase in blood pressure load. Dynamic exercise requires joint movement and change in muscle length and is associated with rhythmic contractions producing a relatively lower intramuscular force compared to static exercise. It is graded (A to C) by estimated percentage of maximal oxygen uptake achieved and typically results in greater increase in cardiac output; these two broad types are a continuum and exercise activities most often involve a combination of both components at differing degrees.

Exercise intensity is often graded in ‘METs’, standing for ‘metabolic equivalent of task’, a measure of energy expenditure as a factor of estimated energy expenditure at rest whereby 1 MET is the energy expenditure at rest for example when sitting awake and is estimated to be equated to oxygen consumption of approximately 3.5 mL per kilogram per minute [18]. It can be used to grade fitness according to the level of intensity a participant can reach.

### 2.2 Benefits of Exercise

Exercise has been observed to have a ‘dose-dependent’ effect on cardiovascular health outcomes. Those who undertake larger volumes of regular exercise achieve greater benefits [19, 20]. The incremental benefits conferred by exercise vary according to baseline level of activity, with the greatest effects per volume increase potentially achieved in those who are inactive, and gradually lower relative gains as exercise volume increases [19].

Minimum recommended exercise levels are 150 minutes per week moderate intensity or 75 minutes per week vigorous intensity aerobic exercise in current international cardiology guidelines [12, 13, 14, 21] (see Table 1, Ref. [13, 21]). These are minimum levels however and research suggests that even greater benefit can be achieved with more exercise and that optimal benefit may be achieved at up to 3–5× minimum recommended levels, though the relative gains are higher in those that are unfit than the fit [2, 13, 22, 23]. European Society of Cardiology (ESC) guidelines recommend a gradual increase to twice minimum levels for additional health benefits [12]. Even low levels of exercise are demonstrably better than none at all [2, 13, 23]. Undertaking resistance exercise on two or more days per week, in addition to aerobic exercise activity, is also advised for additional benefit [12, 13].

**Table 1.** **Exercise recommendations, types and grades of exercise [13, 21]**.

**Minimum Exercise Recommendations**			
150-minutes of moderate-intensity or 75-minutes of vigorous-intensity aerobic exercise per week, divided over 3–5 days			
**Types of Exercise**			
**Types**	**Description**		**Examples**
Aerobic/Endurance/’Cardio’	Activity involving large muscles move for a sustained period of time and that improves cardiorespiratory fitness		Running, Cycling, Aerobics
Muscle-strengthening	Use of muscle force to produce limb movements that train strength, power, endurance and mass of muscle		Weight-lifting, Resistance training
Bone-strengthening	Activity in which force is exerted on bones that may have consequence of promoting bone growth and strength		Running, Skipping
Balance Exercises	Exercises that test participants’ ability to maintain a position against internal and external forces whilst stationary or moving		Yoga, Pilates
Multi-component	An activity that combines elements of multiple activity types		Dancing, Basketball, Rowing
**Grading Intensity of Exercise**			
**Intensity**		**Examples**	
Mild (<3 METS)		Slow walking	
Moderate (3–5.9 METS)		Walking at 4.0–6.4 km per hour, Yoga, Weight-lifting, Volleyball	
*Increased heart and breathing rate but able to hold conversation*			
Vigorous (6.0 METS or more)		Running (>9 km per hour), Football, Basketball	
*Only able to speak a few words at a time*			

MET, metabolic equivalent of task, whereby 1 MET is equal to the resting metabolic rate or energy expenditure when awake at rest, e.g., when sitting. N.B. Graded intensity of exercise will vary from these examples depending on the manner in which performed and certain activities therefore can be of different intensities.

**Fig. 1.**
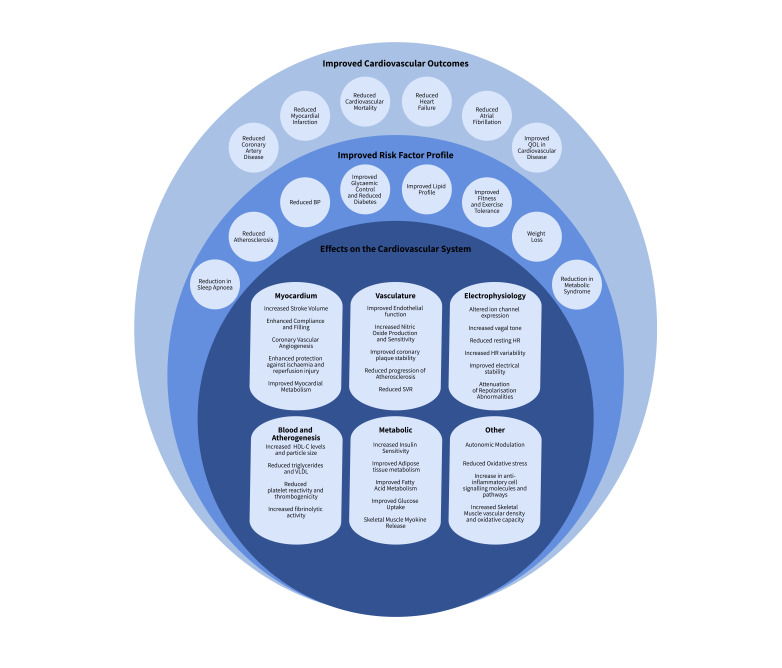
**The beneficial effects of exercise on the cardiovascular system, risk factors and cardiovascular outcomes [1, 4, 7, 10, 11, 27, 28, 29, 30, 31, 32, 33, 34, 35, 36, 37, 38, 39, 40, 41, 42, 43, 44]**. Abbreviations: HDL-C, high-density lipoprotein; HR, heart rate; LV, left ventricle; QOL, quality of life; SVR, systemic vascular resistance; BP, blood pressure; VLDL, very low-density lipoprotein.

Exercise improves cardiorespiratory fitness (CRF), which is a predictor of longevity and inversely associated with cardiovascular events and mortality across different genders, ethnicities and ages [1, 24, 25, 26] (see Fig. 1, Ref. [1, 4, 7, 10, 11, 27, 28, 29, 30, 31, 32, 33, 34, 35, 36, 37, 38, 39, 40, 41, 42, 43, 44]). In a study of over 4000 participants who underwent cardiopulmonary exercise testing (CPEX) to determine baseline CRF, higher baseline CRF as measured by peak oxygen consumption (V02-max) was associated with lower all-cause, cardiovascular and cancer mortality [24]. A 1-MET improvement in baseline CRF equated to a 16% reduction in cardiovascular mortality.

In addition to reducing mortality, exercise is associated with reduced incidence of coronary artery disease (CAD) and myocardial infarction (MI) [45, 46, 47], reduced risk of developing heart failure [48] and reduced burden of arrhythmia [49]. Exercise also improves outcomes through its effects on conventional risk factors for atherosclerosis, including hypertension, diabetes mellitus and dyslipidaemia [8, 9, 27, 46]. In addition, it reduces chronic inflammation [50] and has beneficial effects on autonomic activity resulting in promotion of parasympathetic activity and restoration of autonomic balance [28, 29, 51]. Such health benefits of exercise have been seen to be achieved independently of any consequent reduction in body mass index and obesity [52]. Large meta-analyses have shown that increased levels of exercise are inversely associated with cardiovascular events [53, 54], with one showing that those engaging in 150 minutes per week moderate exercise activity had a 14% reduction in the risk of incident coronary disease [53].

Regular exercise results in chronic reductions in baseline systemic vascular resistance due to enhanced vasodilation mediated by enhanced endothelial nitric oxide and prostacyclin production and increased sensitivity to endothelial nitric oxide synthase [55, 56]. Autonomic modulation and reduction in renin activity and inflammation are also likely to contribute to the blood pressure (BP) lowering effect of exercise [9, 28, 57].

Aerobic exercise is effective in the prevention and treatment of hypertension [9, 58, 59]. A meta-analysis of randomised intervention trials of exercise on BP showed that aerobic or endurance training 3–5 times per week for 30–60 minutes at approximately 40–50% maximal performance was effective in lowering BP and associated with an average reduction of 3.4/2.4 mmHg [59]. A further follow-up meta-analysis confirmed that exercise training reduces resting and daytime BP and found that the BP-lowering effect may be more pronounced in those with hypertension [9]. Additionally, treadmill-based exercise programmes and heated water-based exercise training have been found to be effective in lowering BP in patients with resistant hypertension [10, 60]. Regular dynamic resistance training has also been found to be beneficial in reducing blood pressure over time [61], and is recommended as an adjunct to aerobic training in current guidelines [12].

Exercise also leads to weight loss, reduced body fat and improvements in lipid profile [9]. Though a number of studies have found exercise to be associated with improvements in high-density lipoprotein (HDL-C) levels, findings related to absolute low-density lipoprotein (LDL-C) levels are more inconsistent [27, 62]. For example, in a study of patients with dyslipidaemia, participation in a regular aerobic exercise programme, which varied in amount and intensity according to group, was found to positively impact on the lipoprotein profile, with reductions in small particle LDL-C without affecting overall LDL-C concentration, increase in HDL-C particle size and concentration, and reductions in very low-density lipoprotein and triglyceride concentrations, with higher amounts of exercise showing enhanced effects [27]. Another study found that in healthy young adult males, a 12-week moderate-intensity mixed strength and endurance exercise programme increased HDL-C by 6.6% and decreased LDL-C by 7.2%. Apolipoprotein A1 and B levels were also increased and decreased respectively, and cholesterol efflux capacity was improved [62]. Cardiac rehabilitation programmes incorporating exercise amongst other lifestyle interventions have been seen to improve lipid profile, with reductions in LDL-C, triglycerides, total cholesterol and improvements in HDL-C [63].

Regular exercise also reduces the incidence of type 2 diabetes [8], reduces insulin resistance [9] and may improve glycosylated haemoglobin levels in those with pre-existing diabetes [11]. For example, in a prospective cohort study of over 20,000 United States (US) male physicians, a self-reported frequency of exercise activity vigorous enough to work up a sweat was associated with an age-adjusted relative risk of developing diabetes of 0.77 for once weekly exercise versus no exercise and 0.58 for five-times weekly or more [8]. A meta-analysis of studies evaluating the impact of physical activity on fasting glucose and glycosylated haemoglobin (HbA1c), found that for patients with type 2 diabetes and pre-diabetes, an increase in physical activity of 100 minutes per week was associated with an average change in HbA1c of –0.16% [11]. Additionally, long-term exercise may also mitigate some of the effects of ageing on the cardiovascular system, slowing arterial stiffening and the progression of vascular disease [64] and counteract reductions in left ventricular compliance [65].

Exercise training is beneficial across age spectrums, including the elderly, some of whom may be prone to physical inactivity, deconditioning, frailty and increased risk of cardiovascular disease. Adherence to exercise recommendations is as important in this age group than any other. Studies in older individuals show that exercise effectively improves CRF [66, 67]. For example, in a study of 12 healthy sedentary older adults, a 1-year programme of endurance exercise training induced favourable effects on arterial function and improved fitness, as measured by VO2-max [66]. Muscle weakness can be a feature of ageing and resistance exercise can be beneficial to improve strength and functional performance [68]. Furthermore, comprehensive cardiac rehabilitation programmes in the elderly have been shown to reduce morbidity and mortality. In an evaluation of over 600,000 US Medicare patients aged 65 or older and hospitalized for coronary disease or revascularisation, mortality was 21 to 34% lower in cardiac rehabilitation participants than non-participants [69], and a separate study of elderly US cardiac rehabilitation patients found there to be a dose-response relationship between the number of sessions attended and risk of mortality or MI [70].

Participation in resistance exercise is associated with reduced mortality and has an additive effect to the benefits of aerobic exercise in a training regime [71]. Studies comparing aerobic training to regimes involving resistance training suggest that both forms of exercise are beneficial but that aerobic training in particular improves CRF [72, 73] and that the addition of resistance training in a combined regime produces greater benefits to strength, body composition and risk factor profile [72, 74]. Combining both aerobic and resistance exercise in a fitness regime is important to maintaining good health and fitness [12, 74]. A meta-analysis involving over 500 patients found that combined training in patients with coronary artery disease was associated with greater reductions in body fat and improvements in strength and peak work capacity compared to aerobic training alone [74].

Despite the excellent health benefits of exercise, many do not meet recommended exercise levels and physical inactivity is prevalent worldwide [75, 76]. Engaging in physical activity and exercise can be challenging, especially as much of modern society is geared to facilitate sedentariness, which is itself an independent risk factor for increased mortality [77]. An evaluation of self-reported physical activity and exercise habits in nearly 20,000 adults aged 18 to 64 in Europe found that over a quarter of participants were physically inactive [75]. Epidemiological studies show that physical inactivity is associated with substantial global disease and consequent economic burden [78, 79]. Physical inactivity has been estimated to contribute to 7.2% of global all-cause mortality and 7.6% of cardiac mortality [78]. Therefore increasing exercise uptake and reducing sedentary behaviour as part of primary and secondary prevention are crucial to reduce the burden of cardiovascular disease and mortality, especially given the growing expected epidemic of obesity and other cardiovascular risk factors [12, 80]. Exercise in its many forms can be a cheap and highly accessible mode of primary and secondary prevention. Individual and population-based strategies and recommendations to promote exercise and physical activity for the maintenance of health are increasingly important [13]. In the cardiovascular population, cardiac rehabilitation is underutilised worldwide [81] despite its substantial benefits [82] and improving referral rates and uptake may help to improve outcomes.

## 3. Physiological Adaptation to Exercise

Exercise results in repeated, transient haemodynamic changes, often involving increased pressure and/or volume load, with increased preload and afterload. Cardiac output must be increased to meet the demands of sports, especially at high levels [83]. Sustained, repeated exercise training over time results in cardiovascular remodelling to meet these demands. This effect has been well studied in athlete populations. Due to the amount and intensity of exercise involved, athletes in particular may exhibit structural, functional and electrical changes, such as ventricular hypertrophy, increased ventricular mass, increased chamber volumes, enhanced filling and increased stroke volume, however some who participate in recreational exercise may reach training levels approaching that of athletes.

Electrophysiological changes that occur as a result of long-term exercise may manifest on the electrocardiogram (ECG) as sinus bradycardia, first-degree atrioventricular block, high-voltage QRS complex, incomplete right bundle branch block, early repolarisation and high-voltage T waves [84]. These are often a result of increased vagal tone or a reflection of underlying structural adaptations.

Exercise with a high static component may be associated with sustained blood pressure changes but minimal changes in heart rate and ventricular volumes. Dynamic exercise is associated with greater changes in heart rate and ventricular volumes. It was previously posited that long-term aerobic, high dynamic activity exercise led to biventricular dilatation with minimal eccentric hypertrophy, and that resistance or static training led to marked concentric left ventricular hypertrophy (LVH) with preserved ventricular volumes [85]. However more recent studies suggest that those undergoing strength training often display very minimal remodelling and that increase in left ventricle (LV) mass is proportional to the increase in chamber size regardless of the type of activity [83, 86, 87, 88].

Athletes usually exhibit a volume increase of all 4 chambers [89]. A study of 381 patients undergoing cardiac magnetic resonance scanning (CMR) found that athletes (who exercised for an average of 17 hours per week) in all sporting categories demonstrated balanced cardiac adaptation, with preserved right ventricle/left ventricle (RV/LV) volume ratios [88]. Both those undertaking predominantly high static component and those performing predominantly high dynamic component training showed balanced increase of the ventricular volumes and wall mass, though those performing high static-low dynamic training showed little change in ventricular indices compared to non-athlete controls. Those undertaking high dynamic-high static activities exhibited the greatest change in volume and mass, with relatively more increase in LV wall mass. Biatrial dilatation on CMR has been observed in male triathletes [90] compared to controls and a study of female volleyball players found that biatrial volume, as assessed by echocardiography, increased after a 16-week period of intensive training [91].

A study of serial left ventricular echocardiographic indices in 286 professional cyclists showed that 75% exhibited increased LV end-diastolic diameter and 8.7% had increased wall thickness, though this was always <15 mm and increased wall thickness was rare in the absence of LV dilatation [43]. 20% of the athletes had reduced LV ejection fraction (less than 52%). RV volumes may also increase in response to chronic exercise. In a study of 33 male master endurance athletes with an average training history of 29 years, RV end-diastolic volume assessed by CMR was significantly higher than in controls, but there was no difference in RV ejection fraction [92]. Furthermore, in a study of olympic athletes, approximately one-third exhibited RV dilatation above the threshold for a minor diagnostic criterion for arrhythmogenic right ventricular cardiomyopathy (ARVC) according to Task Force criteria, but the presence of other pathological features was rare [93]. Such findings highlight the importance of accounting for an individual’s exercise history in the interpretation of potentially abnormal findings.

Features of physiological cardiac adaptation may vary according to the type, frequency, intensity and duration of training activity involved [83, 88]. They vary between individuals and are also dependent on environmental factors and individual characteristics, such as sex, ethnicity and age [94, 95]. For example, in studies evaluating ethnic differences in cardiac adaptation, both male and female athletes of African and Afro-caribbean descent have a higher prevalence of left ventricular hypertrophy and ECG repolarisation anomalies than Caucasian athletes, though female African and Afro-caribbean athletes rarely display LV wall thickness greater than 13 mm [96, 97, 98]. Caucasian female athletes rarely have LV wall thickness above 11 mm [97].

Based on a study of athletes participating in a United Kingdom (UK) screening programme, female athletes are less likely than males to exhibit ECG features of LVH, prolonged QRS duration or inferior T wave inversion during screening, though more commonly have anterior (V1-3) T wave inversion [95]. In the same study, female athletes had on average smaller absolute LV size, wall thickness and mass than males. Of those participating in dynamic or endurance sporting activities, females were more likely to exhibit eccentric left ventricular remodelling (defined as an increase in chamber size without increase in wall thickness or mass) and less likely to exhibit concentric remodelling (increase in wall thickness without increase in mass) or concentric hypertrophy (increase in wall thickness and mass) compared to male athletes [95]. Female athletes may also exhibit larger indexed RV (right ventricle) dimensions on echocardiography compared to males, according to a study of over 1000 olympic athletes competing in a variety of sporting disciplines [93].

The changes that result from cardiac adaptation to exercise are often referred to as the ‘athlete’s heart’. Many of these changes show considerable overlap with appearances in early or mild forms of cardiomyopathy (such as hypertrophic cardiomyopathy (HCM) or ARVC), and distinguishing between physiological and pathological can be challenging. In cases that pose a dilemma in terms of differential diagnosis, knowledge of what can be considered to be a normal ECG or imaging feature in athletes is crucial [99, 100, 101, 102]. A holistic clinical assessment which includes a detailed history, type, duration and intensity of exercise activity, cardiac symptoms and family history is extremely useful to make a correct diagnosis. This in conjunction with the use of advanced imaging and ECG can often help to accurately identify true pathology from athletic adaptation.

As far as the 12-lead ECG is concerned, the international criteria for ECG interpretation in athletes [84] can aid the differentiation of abnormalities from normal variants. Echocardiography and CMR can provide detailed information on morphology and function, with the latter providing insights on the possible presence of myocardial fibrosis that may suggest cardiomyopathy.

For example, LVH with wall thickness more than 16 mm in male athletes is more likely to result from HCM [103]. Where there is borderline LV wall thickness in the ‘grey zone’ of 12 to 16 mm, a finding of small left ventricular cavity size (<54 mm), diastolic dysfunction (reduced e’ velocity on tissue doppler) on echocardiography and ECG features consistent with HCM are useful indicators of HCM, whereas those with athlete’s heart are more likely to have larger LV cavity size, normal diastolic function, absence of diffuse T wave inversion on ECG and absence of family history [104]. Furthermore, global longitudinal strain on echocardiography may be reduced in HCM but is expected to be normal in athlete’s heart [105]. CPEX can also be used to help differentiate and a finding of peak oxygen consumption >50 mL/kg/minute or >120% predicted favours physiological adaptation rather than HCM [106]. CMR can identify abnormalities such as focal hypertrophy or late gadolinium enhancement, in some patients with HCM, which are not features of physiological adaptation [107]. As described above, females exhibit a smaller magnitude of increased wall thickness in response to exercise training and therefore LV wall thickness greater than 11 mm in Caucasian athletes or 13 mm in African/Afro-Caribbean athletes should arouse suspicion [96, 97]. If the distinction remains unclear, a period of detraining can be recommended with features of athlete’s heart, but not changes associated with HCM, expected to regress after several months [108, 109, 110].

## 4. Sudden Cardiac Death in Athletes

Despite the considerable benefits of regular exercise, apparently healthy athletes may die suddenly. SCD is a major contributor to cardiovascular death in the general population, with estimated incidence in the US over 500 per million each year (1:2000) [111] and is often related to atherosclerotic disease.

Several studies have reported on the epidemiology of SCD in athletes [112, 113, 114, 115, 116]. There is an extreme variability in terms of annual incidence in the various studies and these differences are mainly due to the different methodologies used. Methods of recording SCD events are many (some studies rely on media reports or insurance claims, others on national or regional registries) and differ among studies as well as the approaches to the post-mortem investigations aimed at finding the cause of death. Furthermore, some studies include only SCDs, while others also include sudden cardiac arrests (SCA). The estimated incidence of exercise-related SCD varies according to reports, but ranges from 1:40,000 to 1:300,000, depending on the population, study methodology and definitions of SCD used [112, 113].

In those with an underlying arrhythmogenic cardiac disease, the physiological stressors resulting from intense exertion, including dehydration, acid-base disturbance, electrolyte derangement and catecholamine surges may result in potentially fatal arrhythmias. Risk may be increased in certain populations and with certain sporting activities. For example, a study on college athletes in the United States showed that the overall incidence of SCD was 1:53,703 athlete-years, but significantly higher (1:5200) athlete-years in basketball players [113].

A diverse spectrum of diseases is implicated in SCD, with variable prevalence dependent on the demographics of the victims and the circumstances of death. The majority of SCDs are attributable to atherosclerotic coronary artery disease and generally manifest in individuals in the 4th decade onward [111]. The primary cardiomyopathies and the ion channelopathies are the predominant causes of SCD in the young (<35 years) [114, 115]. Autopsy is an essential first diagnostic step to guide clinical evaluation of surviving relatives toward inherited structural diseases or primary arrhythmogenic syndromes [117, 118].

**Fig. 2.**
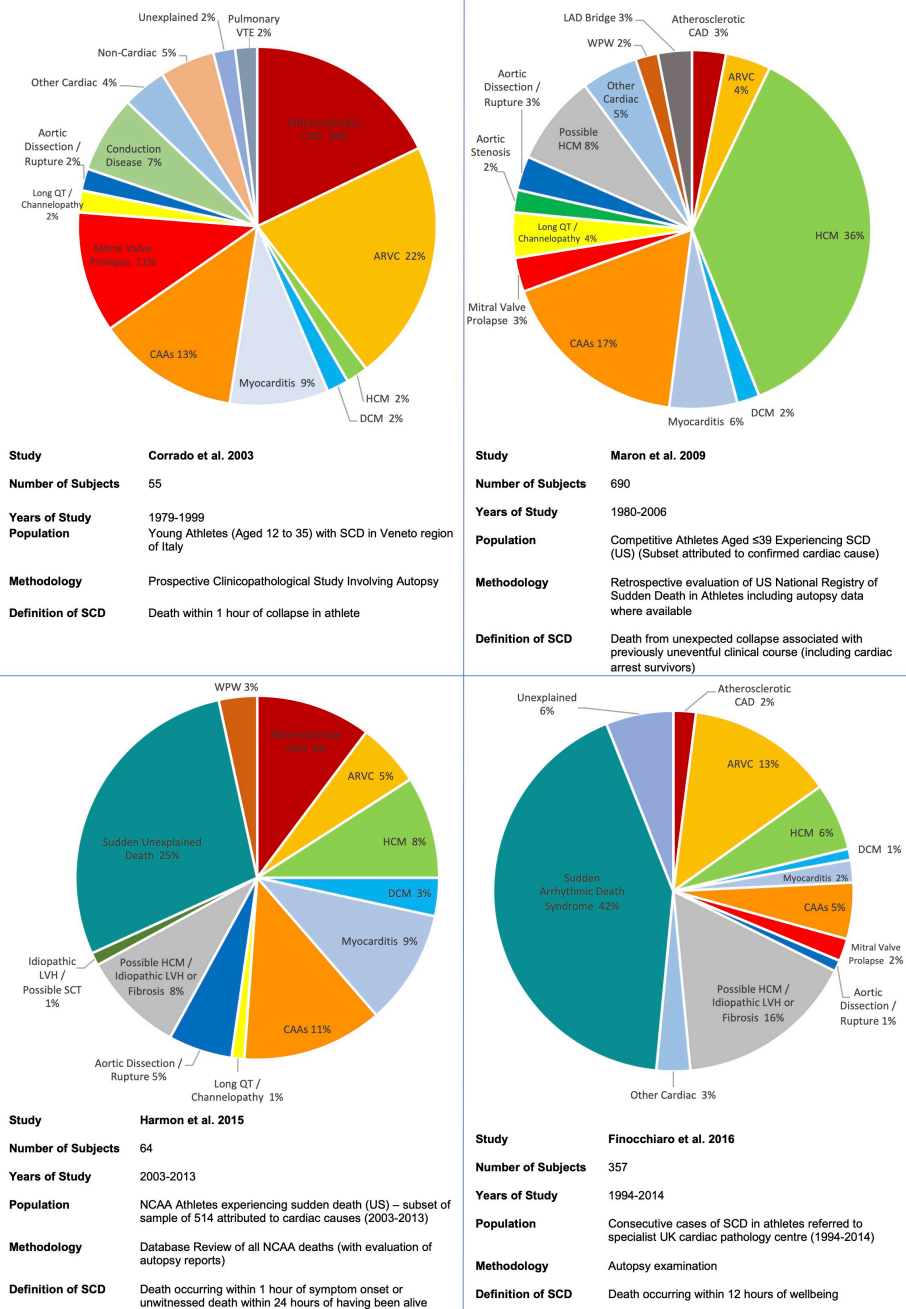
**The causes of sudden cardiac death in four seminal studies, showing a variation in aetiologies depending on the populations, study methodologies and definition of SCD [113, 114, 115, 116]**. Abbreviations: ARVC, arrhythmogenic right ventricular cardiomyopathy; CAAs, coronary artery anomalies; CAD, coronary artery disease; DCM, dilated cardiomyopathy; HCM, hypertrophic cardiomyopathy; LAD, left anterior descending artery; LVH, left ventricular hypertrophy; NCAA, National Collegiate Athletic Association (US); SCD, sudden cardiac death; SCT, sickle cell trait; US, United States; VTE, venous thromboembolism; WPW, Wolff-Parkinson-White syndrome.

Numerous studies have been conducted to elucidate the underlying aetiologies of SCD in athletes, with significant variability in terms of results (see Fig. 2, Ref. [113, 114, 115, 116]). HCM was traditionally considered the most common cause of SCD in young athletes in the United States according to the studies by Maron *et al*. [114], which showed that this condition accounted for up to 36% of the cases. Recent studies have reported different results. A study by Corrado *et al*. [115] showed that, in the Veneto region in Italy, ARVC was the most common cause of SCD in young athletes (22% of the cases). Eckart *et al*. [119] examined 902 cases of SCD in active military personnel from the Department of Defense in the United States. In young individuals (<35 years of age), the heart appeared structurally normal at the post-mortem examination in 41% of cases. HCM accounted for only 13% of cases [119]. Harmon *et al*. [113] reported that a structurally normal heart at the post-mortem examination, suggestive of sudden arrhythmic death syndrome (SADS) was the most common finding (25%) in 64 cases of college athletes who died suddenly. Coronary artery anomalies (CAAs) were the second most frequent cause (11%) and HCM accounted for 8% of the cases [113].

Finocchiaro *et al*. [116] described a cohort of 357 athletes who died suddenly in the United Kingdom where the post-mortem examination was performed by an expert cardiac pathologist. The most common finding was a normal heart at the post-mortem examination, in keeping with SADS (42% of cases).

Individuals that experience SCD are often asymptomatic prior to death [120, 121]. Identifying those at risk is important so that potential harm derived from intense exercise can be avoided, which may be through sporting restrictions, pharmacological therapy or use of implantable cardioverter defibrillator device. Pre-participation screening (PPS) can help to identify those at risk. This can take the form of a structured history and examination, possibly including ECG and imaging tests to identify common electrical and structural abnormalities. The use and appropriate scope of PPS is controversial and debated widely. Opponents highlight that exercise-related SCD is rare and that there may be an unacceptable number of false positive diagnoses during PPS, which lead to unfair disqualification from sports and the associated personal and financial costs [122]. However, others argue that SCD is potentially preventable and given its disproportionate effect on younger individuals, exercise-related SCD may result in many life-years lost despite being rare, therefore justifying recommendations for PPS in athletes [123, 124, 125, 126]. A nationwide screening programme in Italy has been used effectively with an accompanying reduction in the incidence of SCD [123]. Additionally, as experience with PPS grows, targeted diagnostic criteria can improve specificity and lower false positive rates [84].

Education to increase awareness of the risks of SCD, and the use of treatment such as automated external defibrillators is important in helping to reduce the burden of exercise-related SCD. Furthermore, though SCD risk may be acutely increased in certain individuals, habitual regular exercise reduces the overall risk of SCD in a population [127]. A meta-analysis of studies assessing habitual physical activity and risk of SCD, including more than 130,000 participants, found that those with a high level compared to a low level had reduced risk of SCD, with relative risk of 0.52 of SCD in those with the highest level of physical activity [127]. Exercise is safe in the majority and should continue to be encouraged.

## 5. Exercise in Patients with Cardiovascular Disease

Patients with cardiovascular disease may have some hesitancy towards undertaking exercise and uncertainty regarding what and how much is safe. Exercise is most often safe in those with cardiovascular disease and moderate exercise should be promoted in all, though some patients need further risk stratification or specific restrictions. However, competitive sports may be discouraged due to the potential increased risk of harm in susceptible patients. The positive benefits of regular exercise extend beyond primary prevention of disease and can reduce disease progression and improve outcomes in those with cardiovascular disease. It is important to provide suitable guidance, reassurance and advice to patients so that they may access the plentiful benefits of exercise whilst minimising any risks involved.

Many patients will benefit from a structured exercise programme to facilitate exercise that combines an individualised assessment of fitness, exercise guidance, lifestyle advice and support. This may be done via a cardiac rehabilitation programme, which is a highly cost-effective intervention for cardiovascular secondary prevention [128]. Furthermore, the benefits achieved vary according to the type, duration and intensity of exercise. Patients may have varying capabilities and therefore many patients may benefit from a personalised exercise prescription. Exercise may also positively impact on mental state [129] and physical activity greater than 150 minutes per week is associated with higher mental wellbeing scores [130]. Psychological wellbeing may be protective and is associated with a favourable risk factor profile [131, 132]. This is particularly important after cardiac events or in chronic disease as it may help patients cope better and improve motivation in managing their health behaviours and disease.

Recent guidelines on cardiac rehabilitation in secondary prevention provide specific exercise guidance for patients with cardiovascular disease including those with coronary artery disease or acute coronary syndromes, heart failure, those who have undergone cardiac surgery and elderly patients [133, 134]. They focus on an integrated approach to individualised lifestyle interventions including systematic use of a ‘FITT’ prescription for exercise training referring guidance on the frequency, intensity, time and type of exercise performed, and use of personalised exercise goals. Many patients, especially those who are high-risk, will benefit from supervised in-hospital programmes however home-based and remote cardiac rehabilitation programmes have been shown to be effective and safe, with minimal adverse events [135, 136].

Exercise training following myocardial infarction can promote healthy remodelling and recovery of left ventricular function, reduce progression of further coronary artery disease and reduce mortality. In a meta-analysis involving over 14,000 patients following MI or revascularisation, participation in a cardiac rehabilitation programme was associated with a 26% reduction in cardiovascular death and 18% reduction in hospitalisation [137].

For patients with heart failure, cardiorespiratory fitness and exercise tolerance are determinants of quality of life. Exercise training can benefit heart failure patients both with reduced ejection fraction (HFrEF) and preserved ejection fraction (HFpEF) [41, 138]. In patients with HFrEF, exercise training can improve quality of life (QOL) and reduce mortality and hospitalisations [41, 44]. In a trial of over 2300 medically stable HFrEF patients, randomised to standard care plus or minus aerobic exercise training, a supervised exercise programme was associated with significant reductions in composite end points of all-cause mortality or hospitalisation (13% reduction) and cardiovascular mortality or heart failure hospitalisation (15% reduction) [44]. Therefore, exercise training is strongly advocated for heart failure patients in current guidelines [12].

Targeted exercise programmes may also be useful for those with atrial fibrillation (AF). In the recently published ACTIVE-AF trial, 120 patients with symptomatic paroxysmal or persistent AF were randomised to a six-month supervised aerobic exercise programme or standard care alone [139]. The exercise intervention involved supervised weekly/fortnightly interval training sessions with an exercise physiologist, tailored to baseline capacity and adjusted according to heart rate or patient perceived exertion levels (by modified Borg scale). Patients were also provided with a personalised home physical activity programme, and a target to increase physical activity time by approximately 20% per week until they reached 210 minutes moderate to vigorous aerobic activity per week. The standard care group were given two educational sessions on the benefits of exercise and encouraged to perform 150 minutes of moderate exercise but were not given an individualised plan.

At six months, 85% of patients in the exercise intervention group were performing at least 150 minutes per week of unsupervised moderate to vigorous exercise and there were no major adverse cardiac events. Those in the exercise group experienced significant improvements in fitness, as measured by peak oxygen consumption, and had a significantly higher freedom from AF on ECG or ambulatory monitoring than the standard care group (40% vs 20% at 12 months, hazard ratio 0.50) and significant reductions in their AF symptom severity scores, after 6 and 12 months. A supervised and personalised exercise intervention can therefore be effective and adhered to in the management of symptomatic AF, and may be more effective than advice alone. Though such programmes are intensive, should they lead to improved longer-term symptom improvement and reduced hospitalisations amongst other benefits, then they appear to be a powerful intervention across a spectrum of chronic cardiac conditions.

It is recommended that most patients should be supported to undertake physical exercise in line with levels recommended for healthy individuals (150 minutes of moderate-intensity aerobic exercise over 5 days or 75 minutes of vigorous aerobic exercise over 3 days every week). The addition of resistance exercise is advised to further improve fitness, maintain muscle mass and reduce body fat in cardiac patients [13]. For those with diabetes, obesity, well-controlled hypertension or dyslipidaemia, additional resistance training 3 times per week is specifically recommended to reduce cardiovascular risk in recent guidelines [12]. Many cardiac patients, particularly those who are elderly, are at risk of frailty, poor mobility and falls. The addition of targeted strength and balance training to augment cardiac rehabilitation may help to further improve physical function and has been seen to improve functional capacity in heart failure [140, 141] and after cardiac surgery [142].

Patients with cardiomyopathy and primary arrhythmia syndromes may particularly be at increased risk of SCD, and therefore they should be carefully assessed and managed according to their individual risk and desired activity. For patients with cardiomyopathy, exercise guidance is specific to the type, phenotype, and where applicable, genotype (see Table 2, Ref. [12, 143, 144]). Recent European Society of Cardiology guidelines provides updated specific exercise recommendations in these patients [12]. Patients with milder HCM phenotypes may now be considered for high-intensity or competitive sports in the absence of risk factors, but even those with phenotype-negative ARVC are now advised against high-intensity exercise.

**Table 2.** **Restrictions on sporting activity in cardiomyopathy based on 2020 ESC Guidelines on sports cardiology and exercise in patients with cardiovascular disease [12, 143, 144]**.

Condition	Recommendation
Hypertrophic cardiomyopathy (HCM)	∙ Participation in high intensity exercise or competitive sports can be considered on the basis of an individualised expert assessment if no additional risk markers are present
	∙ High-intensity exercise is contra-indicated in the presence of any HCM risk markers (cardiac symptoms, unexplained syncope or history of cardiac arrest, moderately elevated ESC-HCM risk score of 5-year SCD risk (≥4%), resting LVOT gradient >30 mmHg, abnormal BP response to exercise (failure of BP to increase appropriately with exercise) and history of exercise-induced arrhythmias)
	∙ Patients who have risk markers may participate in low or moderate intensity recreational exercise depending on individualised expert assessment
	∙ Genotype positive, phenotype negative HCM patients may be eligible for high-intensity or competitive sport
Arrhythmogenic right ventricular cardiomyopathy (ARVC)	∙ All individuals can be considered for low-intensity exercise
	∙ Patients who have risk markers may participate in low or moderate intensity recreational exercise depending on individualised expert assessment
	∙ High-intensity exercise and competitive sports are not recommended
	∙ Genotype positive, phenotype negative ARVC patients are not recommended to undertake high-intensity exercise or competitive sport
Dilated Cardiomyopathy (DCM)	∙ Participation in high intensity exercise or competitive sports can be considered on the basis of an individualised expert assessment in those who are asymptomatic and if no additional risk markers are present (LVEF <45%, presence of frequent/complex ventricular arrhythmias on ambulatory ECG/exercise testing, LGE on CMR, lamin A/C or filamin C genotype, inability to increase EF by 10–15% during exercise)
	∙ In the absence of limiting symptoms or exercise-induced ventricular arrhythmias, patients may participate in low or moderate intensity recreational exercise regardless of LVEF
	∙ Genotype positive, phenotype negative DCM patients may be eligible for high-intensity or competitive sport, provided they do not have lamin A/C or filamin C genotype
LV Non-compaction Cardiomyopathy (LVNC)	∙ Participation in high intensity exercise or competitive sports can be considered on the basis of an individualised expert assessment if patients are asymptomatic, LVEF ≥50% and frequent/complex ventricular arrhythmias are not present
	∙ Low to moderate intensity exercise can be considered if LVEF 40–49% if there is no syncope and no evidence of frequent/complex ventricular arrhythmias on ambulatory ECG/exercise testing
	∙ Genotype positive, phenotype negative LVNC patients may be eligible for high-intensity or competitive sport, provided they do not have lamin A/C or filamin C genotype
	∙ High-intensity exercise is contra-indicated where symptoms are present, where LVEF <40% and in the presence of frequent/complex ventricular arrhythmias on ambulatory ECG/exercise testing

Abbreviations: ECG, electrocardiogram; ESC, European Society of Cardiology; LGE, late gadolinium enhancement; LVEF, left ventricular ejection fraction; LVOT, left ventricular outflow tract.

In HCM, it is important to evaluate for markers of risk when determining safety for participation in exercise [12, 143], including elevated ESC-HCM risk score of 5-year SCD risk (≥4%). In those with ARVC, high-intensity or competitive exercise is strongly associated with SCD. In a UK autopsy study of 357 SCD victims, over 90% of those whose diagnosis was ARVC had experienced SCD during exertion [116]. Exercise can increase penetrance and expression of the ARVC phenotype in susceptible individuals [145]. It has also been shown to increase heart failure, increase ventricular arrhythmias and worsen survival from the first ventricular tachycardia/fibrillation (VT/VF) event [145]. Therefore, high-intensity and competitive exercise is strongly discouraged in ARVC [12].

In those with long QT syndrome, high intensity exercise is contra-indicated where there has been prior arrhythmogenic syncope or cardiac arrest, and in those with corrected QT (QTc) >500 ms but can be considered in individuals with mild or absent phenotype who are asymptomatic and optimally treated with beta-blocker following individualised expert assessment [12].

Serial follow-up is beneficial, especially for those with cardiomyopathy who exercise regularly, those at increased risk or those who are genotype-positive but without phenotypic features [12, 143]. It is important that any decisions on recommended activity and restrictions are subject to shared decision making, with clear discussion of the risks and documentation in the medical notes.

## 6. Cardiotoxic Effects of Exercise

Whilst moderate exercise has been repeatedly shown to be safe and effective in improving cardiovascular outcomes [13], prolonged high-intensity endurance exercise over many years may have cardiotoxic effects. Very high or extreme volumes of exercise may lead to a reduction in the health benefits or increase in harm, therefore leading to a U-shaped or reverse J-shaped relationship between exercise volume and adverse health outcomes [22], however the level at which this may occur is not fully established. This concept is much debated and other studies have not found evidence of harm at over 10 times minimum recommended activity levels [23, 146].

Long-term athletes may have repeated exposure to the acute stressors of exercise, including mechanical stresses on the heart, hypertensive responses and transient pro-inflammatory state. Over time and at high levels, such repeated exposure may potentially cause deleterious effects despite the benefits of regular exercise. Strenuous and endurance exercise is associated with transient elevations in cardiac biomarkers, including cardiac troponin T (TnT), brain natriuretic peptide (BNP) and d-dimer [147, 148]. A meta-analysis involving studies of cardiac biomarkers post-exercise found that in 4 studies measuring high-sensitivity TnT (hs-cTnT) including 217 patients, 83% had abnormal hs-cTnT immediately post-exercise, with an average change in baseline of 26 ng/L [147]. The reason for and implications of these elevations are unclear, though may result from subtle myocardial necrosis or increased membrane permeability as a result of exercise [149]. A study of 11 patients undergoing CMR acutely post-marathon found no correlation between cardiac biomarker elevations at 6 hours and measures of function, inflammation or fibrosis [150]. Other studies have found that there may be transient increase in RV end-diastolic diameter and reduction in RV ejection fraction immediately post-exercise with subsequent normalisation [147, 148].

**Fig. 3.**
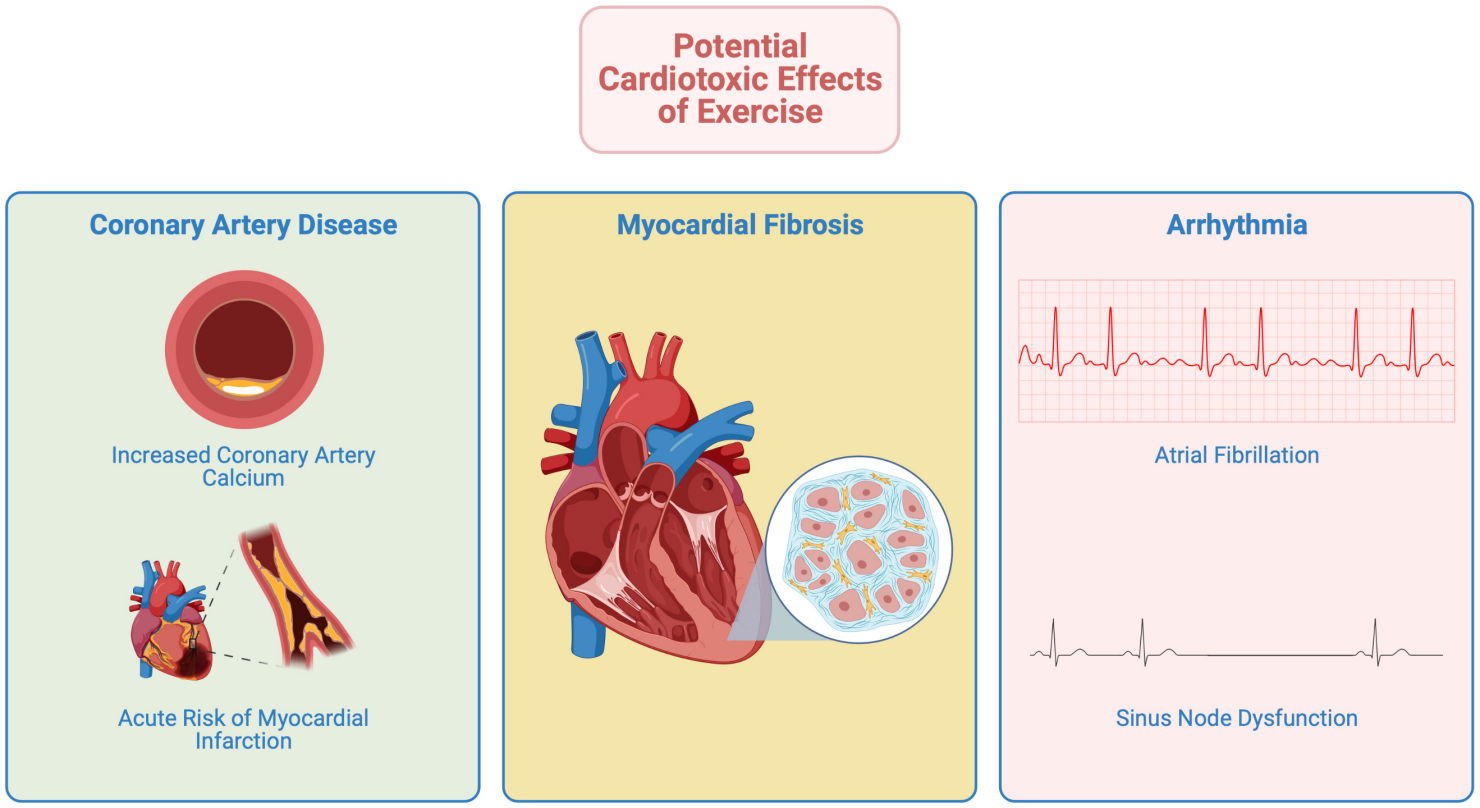
**Potential cardiotoxic effects in those undertaking long-term endurance exercise**.

Certain adverse sequelae have been noted in long-term endurance athletes and this may account for an observed plateau in benefits at very high levels of exercise (see Fig. 3).

### 6.1 Coronary Artery Disease

Prolonged participation in endurance exercise is associated with higher levels of coronary artery calcium (CAC). Whilst the majority of athletes will not experience significant calcification, athletes have been found to have higher CAC scores and amounts of atherosclerosis on CT coronary angiography (CTCA) compared to matched non-athletic controls [151] and very vigorous exercise intensity has been associated with greater CAC and calcified plaque progression in middle-aged and older athletes [152]. However compared to controls, despite male athletes having a greater amount of calcified plaques they had a lower proportion of mixed morphology plaque, which may be more susceptible to plaque rupture [151]. Female athletes did not show a difference in CAC score or plaque morphology compared to controls [151], which could potentially result from a protective effect from higher levels of oestrogen [153]. It is unclear whether the presence of CAC in this setting has a significant negative impact on outcomes. It is possible that calcified plaques observed in the athlete exhibit a different behaviour and prognosis than those seen in the general population, or that athletes have protective mechanisms as a result of adaptation that counteract the presence of calcified plaques [153]. A study of athletes categorised by self-reported exercise dose, found those with the highest exercise dose (>3000 MET-minutes per week) were more likely to have CAC [154] but with no associated increase in mortality for those with high levels of activity and CAC after a decade of follow-up.

The acute effects of exercise may increase the risk of plaque rupture and MI; however among endurance athletes that experience MI or SCD during long-distance events, many do not have signs of plaque rupture [155] since MI in these instances may be due to demand ischaemia in those with coronary stenoses or from coronary spasm [155].

### 6.2 Arrhythmias

Exercise has been shown to have a protective effect on the burden of AF [49]. Physical inactivity and lack of exercise certainly increase the risk of AF and its risk factors [42].

However, there have been ongoing concerns that prolonged high intensity exercise may be a risk factor for the development of atrial arrhythmias, such as AF and atrial flutter, and there may be a U- or reverse J-shaped association between exercise and occurrence of AF with those at low or high extremes of activity level exhibiting increased risk. A number of studies in professional athletes have shown that there is a higher incidence of AF in these athletes than the general population [156, 157]. Those participating in regular, long-term vigorous activity have been observed to have a higher incidence of AF [156, 157]. In a study of over 16,000 men, those who participated in 5 to 7 days per week of vigorous exercise had a 20% increased risk of AF compared to those not undertaking vigorous exercise [156].

Potential mechanisms for this increased risk may include exercise-mediated adaptations to atrial structure and function including atrial dilatation, atrial fibrosis, pulmonary vein stretch, alterations in parasympathetic and sympathetic activity, vagal tone enhancement and inflammation [158, 159, 160].

The incidence of sinus node dysfunction may also be increased in long-term athletes, which is likely mediated through increased vagal tone [83].

### 6.3 Myocardial Fibrosis

Some studies have shown that participation in intense exercise may lead to the development of myocardial fibrosis. This may present in a variety of patterns [89, 151] suggesting different potential mechanisms to exercise-induced fibrosis. Insertion point fibrosis may be consistent with repeated pressure and volume overload, whereas the mechanism for subendocardial enhancement may represent subclinical infarction related to exercise and subepicardial enhancement may represent inflammation or myocarditis [153]. Whilst insertion point fibrosis has been seen in both male and female athletes [89], other studies have observed other patterns of LGE but only in male athletes [151, 161]. In a study of 152 middle-aged and older athletes, 14% of male athletes but no female athletes had late-gadolinium enhancement (LGE) on cardiac MRI scans [151]. These individuals had a higher rate of non-sustained VT. The full implication of an incidental finding of myocardial fibrosis in athletes remains unclear as well as the link with potential adverse outcomes. Tracking biomarkers over time and in response to training regimes may in the future provide a strategy help to identify the risk of developing cardiac fibrosis and other cardiotoxic sequelae in response to exercise. Biomarkers such as ST2 (interleukin-1 receptor-like 1) that have been associated with fibrosis have been seen to be increased in non-elite marathon and ultramarathon runners post-marathon [162].

In summary, a cardiotoxic effect of high intensity exercise has been postulated, with a few studies supporting the notion that the relationship between exercise and health benefits follows a U-shaped curve [22].

## 7. Conclusions

Exercise is a powerful means of improving health, and specifically decreasing cardiovascular morbidity and mortality, partly through an impact on the burden of cardiac risk factors. Regular, moderate exercise is recommended by health bodies worldwide for those with and without cardiovascular disease. Exercise in elite athletes is often accompanied by a plethora of cardiovascular physiological changes. Exercise may be harmful in certain individuals with a predisposed risk to potentially life-threatening arrhythmias and SCD. Despite the many benefits on general and cardiovascular health, exercise may have a cardiotoxic effect especially when it is particularly intense. The higher prevalence of coronary calcifications, myocardial fibrosis and atrial arrhythmias in veteran athletes suggests that exercise taken at the highest levels for years or even decades may result in pathological cardiac changes. Despite these concerns, moderate exercise appears to be safe and effective for most individuals, including those with cardiovascular disease. Appropriate screening of potential subjects at risk and tailoring of exercise advice can help individuals to exercise safely.

## Abbreviations

AF, atrial fibrillation; ARVC, arrhythmogenic right ventricular cardiomyopathy; BNP, brain natriuretic peptide; BP, blood pressure; CAA, coronary artery anomaly; CAC, coronary artery calcium; CAD, coronary artery disease; CMR, cardiac magnetic resonance; CPEX, cardiopulmonary exercise testing; CRF, cardiorespiratory fitness; CTCA, computed tomography coronary angiography; DCM, dilated cardiomyopathy; ECG, electrocardiogram; ESC, European Society of Cardiology; HCM, hypertrophic cardiomyopathy; HDL-C, high-density lipoprotein; HR, heart rate; HFpEF, heart failure with preserved ejection fraction; HFrEF, heart failure with reduced ejection fraction; Hs-cTnT, high-sensitivity cardiac troponin T; L, litre; LDL-C, low-density lipoprotein; LGE, late gadolinium enhancement; LV, left ventricle; LVH, left ventricular hypertrophy; LVOT, left ventricular outflow tract; MET, metabolic equivalent of task; MI, myocardial infarction; NCAA, National Collegiate Athletic Association (US); Ng, nanogram; PPS, pre-participation screening; QOL, quality of life; RV, right ventricle; SADS, sudden arrhythmic death syndrome; SCA, sudden cardiac arrest; SCD, sudden cardiac death; SCT, sickle cell trait; ST2, interleukin-1 receptor-like 1; SVR, systemic vascular resistance; TnT, troponin T; UK, United Kingdom; US, United States; VTE, venous thromboembolism; WPW, Wolff-Parkinson-White syndrome.
